# Frailty and inflammation predict prolonged stay in post-emergency geriatric units: a retrospective cohort study

**DOI:** 10.1016/j.jnha.2026.100801

**Published:** 2026-02-10

**Authors:** Alexandre Cornic, Dan Caraman, Olivier Briere, Jennifer Gautier, Cédric Annweiler, Alexis Bourgeais, on behalf of the BACK-UPUG study group

**Affiliations:** a UNIV ANGERS, Health Faculty, University of Angers, Angers, France; b Department of Geriatric Medicine and Memory Clinic, Research Center on Autonomy and Longevity, University Hospital, Angers, France; c UNIV ANGERS, UPRES EA 4638, University of Angers, France; d Robarts Research Institute, Department of Medical Biophysics, Schulich School of Medicine and Dentistry, The University of Western Ontario, London, ON, Canada

**Keywords:** Post-emergency geriatric unit, Length of stay, Frailty, C-reactive protein, Geriatric care

## Abstract

**Background:**

Post-Emergency Geriatric Units (PEGUs) aim to reduce hospital length of stay (LOS) and prevent functional decline by facilitating earlier discharge.

**Aims:**

This study aim to identify factors linked to prolonged hospitalization to improve patient selection for PEGU.

**Methods:**

In this retrospective study at the University Hospital of Angers (Dec 2022–Feb 2024), 590 eligible PEGU patients were analyzed. LOS was categorized as short (0–5 days) or prolonged (6 or more days). Sociodemographic, clinical, and biological data were assessed using univariate and multivariate logistic regression.

**Results:**

Median age was 88, 62.2% were female, and 69% lived at home; 42% had prolonged LOS. Prolonged stay was associated in univariate analysis with higher Charlson Comorbidity Index (OR: 1.10 [1.02−1.19],*p = 0.015*), elevated CRP > 64 mg/L (2.07 [1.46−2.92], *p < 0.001*), and higher Clinical Frailty Scale (1.79 [1.23−2.59], *p = 0.002)*. The multivariate analysis showed that a CRP levels ≥64 mg/L (OR 1.92 [1.35−2.75], p < 0.001) and a CFS equal to or superior to 7 (OR 1.59 [1.07−2.36], p = 0.022) were associated with prolonged LOS.

**Discussion:**

Frailty and inflammation independently predict longer stays. Limitations include retrospective design and patient exclusions.

**Conclusion:**

Frailty and elevated CRP are key predictors of prolonged PEGU stay. Although causality cannot be established due to the retrospective design and potential biases, these findings may help to better characterize older patients who could potentially benefit from PEGU interventions

## 1 Introduction

Life expectancy is increasing [1] and older patients care represents a challenge for healthcare systems across the world. They have more often comorbidities, frequencies, functional limitations or polypharmacy [2]. As a result, an increase in general practitioner consultations and Emergency Department (ED) visits has been observed. In France, 56% patients over the age of 75 are hospitalized after an emergency visit, compared to 17% for other age groups [1]. For the same cause of hospitalization, older patients stay longer than younger ones in context of pneumoniae [3] and spending a single night in ED increases by 40% mortality among frail older patients compared to those directly admitted to wards in the context of increasingly crowded EDs [4,5]. Moreover, a higher length of stay (LOS) in hospitalization is associated with loss of autonomy, higher mortality rates, and increased readmission rates in older patients [6].

Specialized teams and units have gradually emerged worldwide to optimize patients' healthcare pathways while considering the specificities of an aging and vulnerable population, [[7], [8], [9]]. These include mobile geriatric teams [7,9] and more recently Post-Emergency Geriatric Units (PEGUs). Established in 2002 by the French Regional Health Organization System, these acute geriatric care units are managed by geriatricians and are characterized by very short stays, typically less than one week to prevent iatrogenic complications [10]. By 2013, 18 PEGUs existed in France, mainly within university hospitals [[11], [12], [13]].

In response to the COVID-19 pandemic, Angers University Hospital established a crisis Tension Hospital Geriatric Unit in December 2021, modeled after PEGUs. This unit later evolved into a dedicated 11-bed PEGU providing specialized acute care for older adults. It is staffed by a multidisciplinary team—including a physiotherapist, dietitian, social worker, and a geriatrician available seven days a week. Admissions occur exclusively through the ED. During daytime hours, patient selection is performed by the on-call geriatrician in coordination with bed management; at night, emergency physicians make admission decisions independently. Unlike traditional geriatric services, PEGUs are designed to combine short stays (<6 days) with the aim of preventing iatrogenic functional decline while maintaining patient flow. It is therefore essential to carry out rigorous triage and identify the patient profiles most likely to benefit from this unit. The LOS in hospital is a well-established indicator of hospital performances used by hospital administration [6,14].

The objective of our study is to identify the factors associated with prolonged hospitalization in patients returning home from the PEGU at Angers University Hospital.

## 2 Methods

The BACK-UPUG study is a retrospective observational study conducted in the PEGU, at the University Hospital of Angers (France), from December 2022 to February 29th, 2024 (Clinical Trials ID: NCT06239935). All baseline characteristics were retrospectively collected from patients’ medical records.

### 2.1 Study population

Patients included in BACK-UPUG 2 study were adults aged of 75 years old and older admitted to the PEGU of the University Hospital of Angers, France from December 1 st, 2022, to February 29th, 2024. Exclusion criteria were as follows: patients admitted without going through the ED patients, patients who were transferred to other specific acute medical wards or in rehabilitation ward after their hospitalization, patients who did not return home after being discharged from hospital (institutionalization or admission to a residential care facility) and patients who died during hospitalization.

### 2.2 PEGU unit

The PEGU at Angers University Hospital is a dedicated 11-bed unit delivering specialized acute care tailored to older adults. It operates with a multidisciplinary team comprising a physiotherapist, physical activity educator, dietitian, and social worker. A geriatrician is present seven days a week, including weekends. Admissions are exclusively routed through the ED and direct admissions are not permitted. Patient selection is conducted during daytime hours by the on-call geriatrician in close coordination with the hospital’s bed management service. During night-time hours, however, emergency physicians independently assess and decide on admissions to the PEGU, without geriatric input. In contrast to conventional geriatric wards, the PEGU is structured for short-term hospitalizations (<6 days), with the primary objectives of preventing iatrogenic functional decline and maintaining high patient flow. Consequently, rigorous patient triage is essential. If longer stay is anticipated by the on-call geriatrician patients are excluded from admission. Severe cognitive patients with wandering are excluded due to absence of proper facilities to contain the wandering. When longer hospitalization is indicated, patients are transferred to appropriate downstream care units, depending on bed availability.

The discharge planning to PEGU is carried out according to usual multidisciplinary protocols, as in routine clinical practice.

### 2.3 Characteristics

Sociodemographic data were collected retrospectively for all patients. This included information on the place of residence (at home, in a residential care facility or in a nursing home). In France, a “residential care facility” refers to a non-medicalized housing facility designed for older adults who are largely independent. These residences provide private accommodation combined with optional shared services, but do not offer on-site medical or nursing care. They represent an intermediate housing solution between independent living at home and institutional long-term care facilities such as nursing homes. The degree of independence was determined by the presence of formal help (nurses and home care aides, excluding housekeepers), the use of a walking aid and impaired Instrumental Activities of Daily Living (IADL). Instrumental Activities of Daily Living (IADL) 4 item scale refer to complex daily tasks necessary for independent living: managing finances, using transportation, medication management, and using telephone. Fragility was retrospectively evaluated using the Clinical Frailty Scale (CFS) designed for older patients. This scale includes walking aid use, autonomy, independence, and the presence of formal or informal help. It ranges from 1 (very fit) to 9 (terminally ill). We defined three levels: 1–4, 4–7, and 7–9. C-reactive protein (CRP) values, determined at PEGU admission, were collected and categorized into tertiles, the third tertiles CRP ≥ 64 mg/L has been used in analyses. The Charlson Comorbidity Index, which is a tool predicting 10-year patient survival based on comorbidities (such as heart failure, neurological disorders, pulmonary or hepatic diseases, diabetes, cognitive disorders, or cancer), was also calculated [15].

In the ED, the following data were collected: diagnosis at admission, treating physician referral, and severity criteria (hemodynamic instability defined by systolic blood pressure <90 mmHg [16], respiratory failure (respiratory rate >30/min) [16] or respiratory distress or hypoxemia as determined by blood gas analysis or oxygen requirement >6 L/min; renal failure (increase in serum creatinine > 50% in comparison with usual value) [17]; neurological failure (vigilance disorders or delirium) [18].

Finally, the number of medications (distinct molecules according to the 5th level of the Anatomical Therapeutic Chemical (ATC) classification system) and the use of psychotropic drugs were documented. Cognitive disorders were assessed retrospectively using medical record of the hospitalization due to the absence of cognitive evaluation systematically in PEGU (If MMSE was available, cognitive disorders were assessed using a MMSE score <26/30 and if not assessment of cognitive disorders in medical history was used). LOS, discharge location after hospitalization, and rehospitalization within 30 days were also collected.

### 2.4 Outcomes

We considered any hospitalization at the PEGU lasting 6 or more than 6 days to be a prolonged stay, with the target set for the PEGU being a stay of strictly less than 6 days according to the objectives set for this unit.

### 2.5 Covariates

Potential confounding factors included age and sex. We included in the adjusted model the variable significantly associated with a longer stay or with a *p-value < 0.10* in unadjusted model: CCI, excessive polypharmacy (defined as a use of more than 5 medications per day) and use of psychotropes [19], CRP ≥ 64 mg/L at admission, Clinical Frailty Scale score of 7 or more [20], respiratory failure and renal failure.

### 2.6 Statistical analysis

Patient characteristics are summarized using means and standard deviations or frequencies and percentages when appropriate. Comparisons between patients hospitalized for 6 days or more and those hospitalized for less than 6 days were performed using the Chi-square test, Fisher's exact test, or the Student's t-test (or the Mann–Whitney U test, depending on the normality assessment), as appropriate. Then, logistic regression was used to evaluate associations between confounding factors and LOS. The odds ratio and its 95% confidence interval (CI) and p-value were calculated using logistic regression. Multivariate analysis was performed considering age, sex, and all variables associated with a p-value less than 0.10 in the univariate analysis, and according to the referral from the treating physician, as per our practice. To assess potential selection bias related to excluded patients, baseline demographic and clinical characteristics were compared between included patients and those excluded in this study. To assess the etiology of elevated CRP levels, mean CRP levels according to admission diagnosis were determined. All statistics were performed using SAS® version 9.4 (SAS Institute Inc.), and a p-value less than 0.05 was considered statistically significant.

### 2.7 Ethics

The study was conducted in accordance with the ethical standards of the Helsinki Declaration (1983). No participants or relatives objected to the use of anonymized clinical and biological data for research purposes. Ethics approval was obtained from the Ethics Board of the University Hospital of Angers, France (2024-100). The study protocol was also declared to the National Commission for Information Technology and civil Liberties (CNIL;ar23-0105v0).

## 3 Results

### 3.1 Flow chart (Fig. 1)

**Fig. 1:**
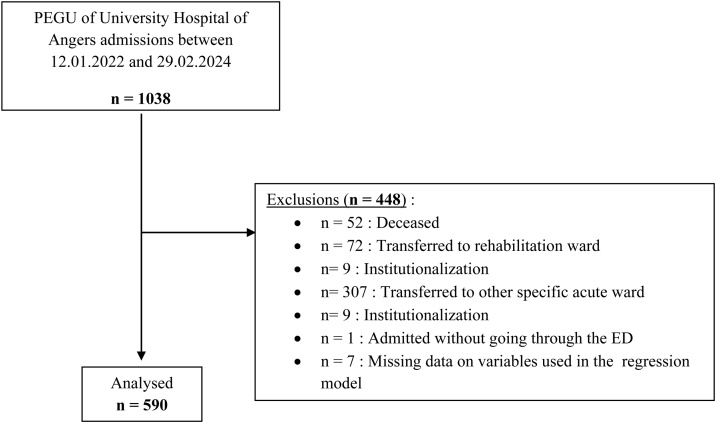
Flow chart. PEGU: Post-Emergency Geriatric Unit. ED: Emergency Department.

From December 1st, 2022, to February 29, 2024, 1038 have been included in the BACK-UPUG 2 study. Out of these patients, 590 participants met the eligibility criteria and were included in the analysis (Fig. 1).

### 3.2 Patient’s characteristics (Table 1)

Table I presents patient characteristics according to the length of their hospitalization at PEGU. Median age was 88 (Interquartile Range (IQR) 83−91), with 62.2% being women and 69% living at home. Sixty-Nine percent (69%) were using walking aids and 42% had prolonged LOS. No significant differences were observed between the two groups regarding severity as assessed by QuickSOFA score.

Patients with prolonged LOS had a median Charlson Comorbidity Index (CCI) equal to 7 vs 6 for the other group (*p = 0.004*). The prolonged LOS group had more frequently CRP levels ≥64 mg/L than the other group (43.6% vs 27.2%, *p < 0.001*). The prolonged LOS group had more frequently Clinical Frailty Scale (CFS) score ≥7 (32.7% vs 21.4%, *p = 0.002*).

**Table 1:** Characteristics and comparison of admitted patients (n = 550) separated into two groups regarding prolonged length of stay or not in PEGU.

		Prolonged length of stay (≥ 6 days)		
	Total (n = 590)	Yes (n = 248)	No (n = 342)	p-value*
General characteristics				
Female gender, n (%)	367 (62.2)	146 (58.9)	221 (64.6)	0.155
Age, years, med [IQR]	88 [83−91]	88 [84−92]	87 [82−91]	0.253
Living place, n(%)				0.852
Home	407 (69.0)	169 (68.2)	238 (69.6)	
Residential care facility	89 (15.1)	37 (14.9)	52 (15.2)	
Nursing home	94 (15.9)	42 (16.9)	52 (15.2)	
Use of walking aid, n (%)	407 (69.0)	180 (72.6)	227 (66.4)	0.108
Impaired IADL (<4/4), n(%) $	452 (77.9)	198 (81.2)	254 (75.6)	0.111
Comorbidities and treatments				
Cognitive disorders n (%)∑	217 (40.5)	103 (44.4)	114 (37.5)	0.107
Charlson Comorbidity Index, med[IQR]	7 [6–8]	7 [6–8]	6 [5–8]	**0.004**
Polypharmacy, n(%) †	483 (81.9)	210 (84.7)	273 (79.8)	0.131
Psychotropes, n(%)	275 (46.6)	122 (49.2)	153 (44.7)	0.284
Patient’s information				
CRP ≥ 64 mg/L, n (%)	201 (34.1)	108 (43.6)	93 (27.2)	**<0.001**
CFS score ≥ 7, n (%)	154 (26.1)	81 (32.7)	73 (21.4)	**0.002**
Hospital readmission, n (%) ‡	75 (12.7)	36 (14.5)	39 (11.4)	0.263
Initial severity and emergency information				
Treating physician referral, n (%)	173 (29.3)	76 (30.7)	97 (28.4)	0.548
Hemodynamic disorders, n(%) ^α^	21 (3.6)	6 (2.4)	15 (4.4)	0.203
Neurological failure, n(%) β	99(16.8)	46(18.6)	53 (15.5)	0.328
Respiratory failure, n(%) £	109 (18.5)	54 (21.8)	55 (16.1)	0.079
Renal failure, n(%) ¥	70 (11.9)	36 (14.5)	34 (9.9)	0.090

CFS: Clinical Frailty Scale.

* Between-group comparisons based on Chi-square test or Mann–Whitney Wilcoxon test, as appropriate.

∑ 54 missing data (9.15%).

† Use of ≥5 medications per day (ie, distinct substances according to the 5th level of Anatomical Therapeutic Chemical [ATC] classification system).

$ Missing data n = 10.

‡ Hospital admission (acute ward or emergency department visit) less than 30 days prior to the Post-Emergency Geriatric Unit admission; α Systolic blood pressure <90 mmHg

β Vigilance disorders or delirium.

£ Respiratory rate >30/min, signs of respiratory distress, hypoxia on arterial blood gas, oxygen requirement exceeding 6 L/min.

¥ Renal failure (increase of creatinine more than fifty percent).

### 3.3 Univariate and multivariate logistic regression (Table 2)

Table II presents univariate and adjusted multivariate analysis. Prolonged stay was associated in univariate analysis with higher Charlson Comorbidity Index (OR: 1.10 [1.02−1.19], *p = 0.015*), elevated CRP > 64 mg/L (2.07 [1.46−2.92], *p < 0.001*), and higher Clinical Frailty Scale (1.79 [1.23−2.59], *p = 0.002*). The multivariate analysis showed that a CRP levels ≥ 64 mg/L (OR 1.92 [1.35−2.75], *p < 0.001*) and a CFS equal to or superior to 7 (OR 1.59 [1.07−2.36]*, p = 0.022)* were associated with prolonged LOS.

**Table 2:** Univariate and multivariate logistic regression showing the odds ratio for prolonged length of stay in PEGU patients (dependant variable) according to patients characteristics (independent variable), adjusted for patient’s characteristics (n = 550).

	Long stay in PEGU			
	Unadjusted model		Adjusted model	
	OR [CI 95%]	p-value	OR [CI 95%]	p-value
Female gender	0.78 [0.56−1.10]	0.156	0.77 [0.53−1.10]	0.149
Age	1.02 [0.99−1.05]	0.210	1.01 [0.98−1.04]	0.403
Charlson Comorbidity Index	1.10 [1.02−1.19]	**0.015**	1.04 [0.96−1.14]	0.332
CRP ≥ 64 mg/L	2.07 [1.46−2.92]	**<0.001**	1.92 [1.35−2.75]	**<0.001**
Psychotropes	1.20 [0.86−1.66]	0.284	1.10 [0.76−1.57]	0.622
Polypharmacy*	1.40 [0.90−2.16]	0.132	1.19 [0.74−1.92]	0.470
CFS score ≥ 7	1.79 [1.23−2.59]	**0.002**	1.59 [1.07−2.36]	**0.022**
Respiratory failure	1.45 [0.96−2.21]	0.080	1.25 [0.81−1.93]	0.321
Renal failure	1.54 [0.93−2.54]	0.092	1.30 [0.77−2.20]	0.326

CI: Confidence Interval; OR: Odds Ratio; CFS: Clinical Frailty Scale; CRP: C-Reactive Protein.

* Use of ≥5 medications per day.

### 3.4 Baseline characteristics of included and excluded patients (Table 3)

Compared with included patients, excluded patients were more frequently male, had high impaired IADL at baseline, presented higher markers of acute severity, including elevated CRP levels (CRP ≥ 64 mg/L) and a higher prevalence of neurological failure or delirium. No significant differences were observed regarding age, comorbidity burden (Charlson Comorbidity Index), frailty level (Clinical Frailty Scale ≥7), polypharmacy, or other acute organ failures.

**Table 3:** Baseline characteristics of analyzed and excluded patients (n = 1038).

			Excluded patients		
	Données manquantes	Total (n = 1038)	Yes (n = 448)	No (n = 590)	p-value*
General characteristics					
Female gender, n (%)		612 (59.0)	245 (54.7)	367 (62.2)	**0.015**
Age, years, med [IQR]		88 [83−92]	88 [83−92]	88 [83−91]	0.137
Charlson Comorbidity Index, med[IQR]		7 [6–8]	7 [6–8]	7 [6–8]	0.175
Impaired IADL, n (%)	23	813 (80.1)	361 (83.0)	452 (77.9)	**0.046**
Polypharmacy, n(%) †	2	853 (82.3)	370 (83.0)	483 (81.9)	0.647
Clinical severity at admission					
CRP ≥64 mg/L, n (%)	17	402 (39.4)	201 (46.6)	201 (34.1)	**<0.001**
CFS score ≥7, n (%)	8	289 (28.1)	135 (30.7)	154 (26.1)	0.106
Hemodynamic disorders, n(%) α		39 (3.8)	18 (4.0)	21 (3.6)	0.700
Neurological failure, n(%) β		215 (20.7)	116 (25.9)	99 (16.8)	**<0.001**
Respiratory failure, n(%) ^£^		200 (19.3)	91 (20.3)	109 (18.5)	0.457
Renal failure, n(%) ¥		136 (13.1)	66 (14.6)	70 (11.9)	0.175

CRP: C-reactive protein; IQR: interquartile range.

* Between-group comparisons based on Chi-square test (or Fisher exact test) or Mann–Whitney Wilcoxon test.

† Use of ≥5 medications per day (ie, distinct substances according to the 5th level of Anatomical Therapeutic Chemical [ATC] classification system).

α Systolic blood pressure <90 mmHg.

β Vigilance disorders or delirium; £ Respiratory rate >30/min, signs of respiratory distress, hypoxia on arterial blood gas, oxygen requirement exceeding 6 L/min.

¥ Renal failure (increase of creatinine more than fifty percent).

### 3.5 Comparison of C-reactive protein levels in patients admitted for infectious versus non-infectious diseases (Table 4)

In Table 4, the most frequent reasons for admission were infectious diseases (31.0%) and falls/mobility disorders (21.0%). Mean CRP levels were highest in patients admitted for palliative care (201.0 ± 21.2 mg/L), but there were only 2 patients, and infectious diseases (104.0 ± 80.3 mg/L. Lower CRP levels were seen in patients admitted for cognitive impairment/behavioral disorders (19.0 ± 19.5 mg/L) and non-infectious neurological disease (27.7 ± 37.0 mg/L).

**Table 4:** C-reactive protein levels according to reason for hospital admission (n = 590).

	n (%)	Mean CRP ± SD (mg/L)
Reason for hospital admission		
Cognitive impairment / behavioral disorders	5 (0.8)	19.0 ± 19.5
Acute confusional state (delirium)	20 (3.4)	53.7 ± 53.2
Falls / mobility disorders	124 (21.0)	46.1 ± 53.5
Pain	13 (2.2)	55.2 ± 65.4
General health deterioration / malnutrition	38 (6.4)	39.2 ± 41.1
Palliative care	2 (0.3)	201.0 ± 21.2
Acute cardiac disease	63 (10.7)	46.7 ± 78.0
Acute pulmonary disease (non-infectious)	27 (4.6)	61.9 ± 96.0
Infectious disease	183 (31.0)	104.0 ± 80.3
Digestive disease (non-infectious)	30 (5.1)	50.8 ± 59.1
Neurological disease (non-infectious, excluding cognitive disorders and delirium)	41 (6.9)	27.7 ± 37.0
Other	44 (7.5)	58.9 ± 75.2

In Table 5, CRP levels were significantly higher in patients admitted for infectious diseases compared with those admitted for non-infectious conditions (p < 0.001).

**Table 5:** Comparison of C-reactive protein levels in patients admitted for infectious versus non-infectious diseases (n = 590).

	n	Mean CRP ± SD (mg/L)	p-value*
Admission type			
Non-infectious disease	407	47.6 ± 63.2	< 0.001
Infectious disease	183	104.0 ± 80.3	

* Given the non-normal distribution of CRP, results are presented using a non-parametric approach (Mann–Whitney U).

## 4 Discussion

In this study, factors associated with prolonged LOS are elevated CRP (superior to 64 mg/L) and CFS equal or higher than 7, independent of all the cofounders accounted in the analysis.

PEGUs are a new model of older patients care characterized by a short LOS, with a highly variable mean LOS in France, ranging from 3.5 to 17 days (average around 7 days) [21]. In the Angers university hospital PEGU the mean LOS is 4.3 days. *Tal Sari et al.* in contrast reported a mean LOS of 6.4 days (close to our results) in an acute geriatric ward [2].

Several reports studied the influence of patient’s characteristics on LOS or other hospitalization outcomes (mortality or 30-day readmission), particularly in the older population [[22], [23], [24], [25], [26]]. Few studies had focus on PEGUs outcomes specifically and even fewer studied on patient related factors influencing LOS in these specific units [10,11,27]. Conflicting evidences remain in explaining the risk factors associated with prolonged hospital stays among older patients. Most cited and consensual patient’s factors in classical geriatric unit are: dementia, confusion, polypharmacy, comorbidity, frailty, severity and infectious disease (as admission diagnosis) [[22], [23], [24],^26^].

In our study, cognitive status was not associated with prolonged LOS. This could be explained by the refusal of patients suffering from inappropriate motor disorders during regulation by the geriatrician. The main reason is the lack of adaptation of the PEGU’s facilities to these disorders. *Tal Sari et al.* report that cognitive status was not influencing by prolonged LOS either [2]. However, other reports suggest that dementia is associated with prolonged LOS in geriatric units with mean longer stay (around 10 days) [28]. Delirium has been associated with prolonged LOS. Delirium in our study has not been associated with prolonged LOS probably due to inconsistency in reporting confusion with the retrospective design. In addition, regulation by the PEGU coordinating geriatrician may result in non-confused patients being given priority, based on bibliographic data and experience of coordinating geriatricians.

In our study, polypharmacy (defined as 5 or >5 medications) was not associated with prolonged LOS contrary to some reports [2,29]. *Vetrano et al*. report that excessive pharmacy (>10 medications) patients has a 3-fold risk of prolonged LOS (>10 days) whereas polypharmacy does not [24]. Moreover, the high prevalence in our population reduces the likelihood of detecting a small effect.

Concerning the infectious disease and biological markers, such as our study, *Brown et al.* observed that an elevated CRP was independently associated with prolonged hospitalization [30]. This is in line with our study, in which a high CRP level remains significant in the multivariate analysis. *Brown et al.* calculated the mean CRP level in 3 distinct groups according to patient discharge. In our study, the analysis is based on CRP tertiles. This also corresponds to studies focusing on infectious diagnoses at admission (pneumonia or urinary tract infection) that were associated with prolonged hospital stays. Infectious disease seems to be the main explanation of elevated CRP in the PEGU population as assessed with supplementary analyses. However, the results diagnoses at admission must be interpreted with caution as geriatric patients often present multiple etiologies which explain the hospitalization and we only reported the main explication at the entry in PEGU despite there might be multiple potential explanation. Fall is often explained by latent infectious disease or other condition in older patients increasing the difficulty to capture the right explanation of the hospitalization in this study [31].

Several studies highlight the link between the degree of frailty and longer hospital stays in older patients. *McDermott et al.* found a positive association between the frailty scale (CFS) and LOS in a subacute discharge unit [32,33]. A CFS score of 7 corresponds to complete dependence for personal care, which has important implications for discharge planning in this study population. Patients with this level of frailty often require complex care arrangements, including caregiver support or institutional placement, which may contribute to longer hospital stays and limit the feasibility of early discharge. The Clinical Frailty Scale (CFS) is widely recognized in the literature as a reliable pre-hospital tool that offers a straightforward clinical assessment by integrating comorbidity, disability and cognitive disorders. Thus, that could explain that comorbidity is not associated with prolonged LOS in our study despite other studies which was not using the CFS [22,24]. While CFS integrates cognitive disorders among others elements, our statistical analysis showed that cognitive impairment alone was not a significant predictor in the univariate and multivariate models.

*Toh HJ et al.* found a link between prolonged hospitalization in geriatric unit and disease severity according to the Systemic Inflammatory Response Syndrome (SIRS) [23]. In our study, severity was assessed by the presence of organic failures adapted from the SIRS. For example, the respiratory rate is defined as higher than 20 cycles per minute in SIRS, whereas in our study it is defined as higher than 30 cycles per minute. These different thresholds and low frequency of events might explain the absence of a significant link between each criterion and length of hospital stay.

The influence of general practitioner referral to the ED on LOS was also assessed in our study. Neither the univariate nor the multivariate model found any association with longer hospital stay. Few studies have explored the influence of general practitioner (GP) referrals in ED and non in hospitalization. *Parsonage MT et al.* found that patients with a referral letter from a general practitioner had a longer LOS at ED than those without [34].

Several reports describe that decreasing LOS is not systematically associated with readmission for specific pathology. Specifically in PEGUs there is some evidences that decreasing LOS is not associated with an increased readmission rate [2,10,35]. In our report, this covariate was not significantly associated with short stays, highlighting the need for rigorous triage to identify patients with a profile compatible with short hospitalization unit without risks of readmission for the patient.

The main limitation of this study is its retrospective nature. Data collection based on medical record information was challenged by missing data such as cognitive disorders and presence of delirium, whose diagnose is difficult to establish in the ED and in PEGU. Moreover, this retrospective nature does not allow collecting some confounding factors such as caregiver stress or other factors such as heath car system’s characteristics [36]. Another limitation is the decision to exclude transferred patients to rehabilitation or other wards because of the difficulty in following them after they leave PEGU. This represents almost half of the sample and may have certainly influenced the results. As a result we decided to perform a sensitivity analysis to describe excluded patients and compare these patients. Excluded patients exhibited higher impaired IADL (at baseline), higher CRP levels and neurological impairment, suggesting that patients with the most severe acute presentations were less likely to be included in the analysis. This selection may have led to an underestimation of the association between acute severity and prolonged length of stay. However, the absence of differences in age, comorbidity burden, and frailty indicates that the included population remains representative of the geriatric profile of patients admitted to the Post-Emergency Geriatric Unit.

The main strength of the study is the size of the sample analyzed with extensive covariate collection and adjustment. Also, there are few studies on PEGU, this geriatric short stay unit being recent and rare. This study could contribute to the development of new criteria for selecting patients who are more likely to benefit from this type of unit.

This study paves the way for future research on factors influencing the length of stay in PEGUs, as some questions remain unanswered. As an example this report does not examine patients' quality of life after hospitalization. A follow-up assessment could be implemented to evaluate quality of life in a prospective design. Several factors unrelated to patient characteristics could influence length of stay, such as organizational specifics (unit close to ED) or the involvement of a social worker. A more in-depth report on the subject would be useful in this regard.

## 5 Conclusion

This study reports that frailty (defined by a CFS score >7) and CRP levels may be associated with prolonged hospital stays. Although causality cannot be established due to the retrospective design and potential biases, these findings may help to better characterize older patients who could potentially benefit from PEGU interventions.

## CRediT authorship contribution statement

Conceptualization and study design: Alexis Bourgeais, Olivier Brière, Cédric Annweiler.

Data acquisition: Alexandre Cornic, Dan Caraman.

Data analysis: Jennifer Gautier, Alexis Bourgeais.

Statistical analysis: Jennifer Gautier, Alexis Bourgeais.

Investigation: Alexis Bourgeais, Alexandre Cornic, Dan Caraman, Jennifer Gautier, Cédric Annweiler.

Writing – original draft: Alexis Bourgeais, Alexandre Cornic, Dan Caraman.

Writing – review & editing: Alexis Bourgeais, Alexandre Cornic, Dan Caraman.

## Declaration of Generative AI and AI-assisted technologies in the writing process

The authors assessed the use of tools used to check references, check grammar, spelling and reformulate (implemented logiciel in Word, Zotero and use of deepL). After using this tool/service, the authors reviewed and edited the content as needed and take full responsibility for the content of the published article.

## Funding sources

The authors declared no founding sources.

## Data avaibility statement

The data are available upon request from the authors.

## Declaration of competing interest

The authors declare that they have no known competing financial interests or personal relationships that could have appeared to influence the work reported in this paper.
